# Amino Acid-Mediated Intracellular Ca^2+^ Rise Modulates mTORC1 by Regulating the TSC2-Rheb Axis through Ca^2+^/Calmodulin

**DOI:** 10.3390/ijms22136897

**Published:** 2021-06-27

**Authors:** Yuna Amemiya, Nao Nakamura, Nao Ikeda, Risa Sugiyama, Chiaki Ishii, Masatoshi Maki, Hideki Shibata, Terunao Takahara

**Affiliations:** Department of Applied Biosciences, Graduate School of Bioagricultural Sciences, Nagoya University, Furo-cho, Chikusa-ku, Nagoya 464-8601, Japan; y.amemiya.n.agr@gmail.com (Y.A.); nakamuranao000@gmail.com (N.N.); ikeda.nao1118@gmail.com (N.I.); 2ke7ir9s@gmail.com (R.S.); chaki_chaki9584@yahoo.co.jp (C.I.); mmaki@agr.nagoya-u.ac.jp (M.M.); shibabou@agr.nagoya-u.ac.jp (H.S.)

**Keywords:** amino acid, calcium, mTOR, TSC, Rheb GTPase, calmodulin

## Abstract

Mechanistic target of rapamycin complex 1 (mTORC1) is a master growth regulator by controlling protein synthesis and autophagy in response to environmental cues. Amino acids, especially leucine and arginine, are known to be important activators of mTORC1 and to promote lysosomal translocation of mTORC1, where mTORC1 is thought to make contact with its activator Rheb GTPase. Although amino acids are believed to exclusively regulate lysosomal translocation of mTORC1 by Rag GTPases, how amino acids increase mTORC1 activity besides regulation of mTORC1 subcellular localization remains largely unclear. Here we report that amino acids also converge on regulation of the TSC2-Rheb GTPase axis via Ca^2+^/calmodulin (CaM). We showed that the amino acid-mediated increase of intracellular Ca^2+^ is important for mTORC1 activation and thereby contributes to the promotion of nascent protein synthesis. We found that Ca^2+^/CaM interacted with TSC2 at its GTPase activating protein (GAP) domain and that a CaM inhibitor reduced binding of CaM with TSC2. The inhibitory effect of a CaM inhibitor on mTORC1 activity was prevented by loss of TSC2 or by an active mutant of Rheb GTPase, suggesting that a CaM inhibitor acts through the TSC2-Rheb axis to inhibit mTORC1 activity. Taken together, in response to amino acids, Ca^2+^/CaM-mediated regulation of the TSC2-Rheb axis contributes to proper mTORC1 activation, in addition to the well-known lysosomal translocation of mTORC1 by Rag GTPases.

## 1. Introduction

Cells appropriately regulate cell growth, metabolism, and autophagy in response to environmental cues such as amino acids, glucose, growth factors, and stresses. A key mediator of these responses is a multiprotein kinase called mechanistic target of rapamycin complex 1 (mTORC1), which includes the protein kinase mTOR and two core subunits, Raptor and mLST8. In addition to mTORC1, mTOR can form another complex called mTORC2, which contains Rictor as a defining component, instead of Raptor, and has distinct functions such as regulation of actin organization [1,2,3]. It has been proposed that two signaling axes stimulated by amino acids and by growth factors independently converge on mTORC1 to fully activate mTORC1 [2,3,4]. Amino acids mainly regulate intracellular localization of mTORC1 by Rag GTPases. Rag GTPases are heterodimeric GTPases consisting of RagA or RagB bound to RagC or RagD. Rag heterodimer localizes at the surface of lysosomes through its binding partner, Ragulator complex. Upon stimulation by amino acids, Rag heterodimer is converted to active forms, i.e., GTP-bound RagA and GDP-bound RagC. The active Rags can recruit mTORC1 onto the lysosomal surface. Growth factors regulate Rheb GTPase, a potent activator of mTORC1 localized to the lysosomal surface, although the precise subcellular localization of Rheb remains controversial [5,6,7,8,9]. It has been shown that growth factors promote the GTP-loading of Rheb by inactivating TSC2, a GTPase activating protein (GAP) toward Rheb. Mechanistically, growth factors have been reported to induce inactivating phosphorylation of TSC2, and/or to promote TSC2 translocation from lysosomes to the cytoplasm, leading to activation of Rheb [6,10,11]. In contrast, cellular stresses such as energy depletion and hypoxia activate and promote lysosomal recruitment of TSC2, leading to inactivation of Rheb and thereby of mTORC1 [12].

It was reported that mTORC1 activity can be regulated by the intracellular Ca^2+^ concentration [13,14]. Indeed, treatment of cells with BAPTA-AM, an intracellular Ca^2+^ chelator, resulted in reduced mTORC1 activity [13]. Furthermore, previous studies showed that addition of amino acids, especially leucine, increases intracellular Ca^2+^ concentration through an unidentified mechanism and modulates mTORC1 activity [3,15]. Moreover, calmodulin (CaM) senses an elevated Ca^2+^ concentration and in turn binds to hVps34, a class III phosphatidylinositol-3-kinase, to activate mTORC1. Consistently, hVps34 has been reported to be activated by amino acids [16]. However, the involvement of Ca^2+^/CaM in the activation process of hVps34 remains controversial [17], and detailed mechanism by which hVps34 is activated is still unknown.

From the current view of the proposed mTORC1 activation mechanism, which involves Rags and Rheb, it is reasonable that amino acids are primarily required for mTORC1 activity at lysosomes due to the rate-limiting step for mTORC1 activation [2,3,4]. In contrast, even in the absence of growth factors, when Rheb is thought to be inactivated, amino acids availability can still activate mTORC1 to some extent. Importantly, Rheb is an essential factor for mTORC1 activation, and mTORC1 is unable to be activated in the absence of Rheb [18,19]. Thus, it remains unclear whether amino acids can also promote Rheb activation to appropriately promote the mTORC1 pathway.

Here we show that amino acids activate mTORC1 by modulating the TSC2-Rheb pathway in addition to Rag GTPases. We found that amino acids stimulate extracellular Ca^2+^ entry that leads to a rise in intracellular Ca^2+^ concentration. The elevation of Ca^2+^ contributes to mTORC1 activation and to cellular functions such as promotion of protein synthesis. We revealed that CaM plays a key role linking Ca^2+^ signaling to TSC2 to promote mTORC1 activation. Ca^2+^/CaM interacts with the GAP domain of TSC2 and might thereby regulate its GAP activity toward Rheb. Thus, our findings revealed a new regulatory pathway of amino acid-induced activation of the mTORC1 pathway through the TSC2-Rheb GTPase axis by controlling intracellular Ca^2+^ levels.

## 2. Results

### 2.1. Addition of Amino Acids Induces Ca^2+^ Entry

**Figure 1:**
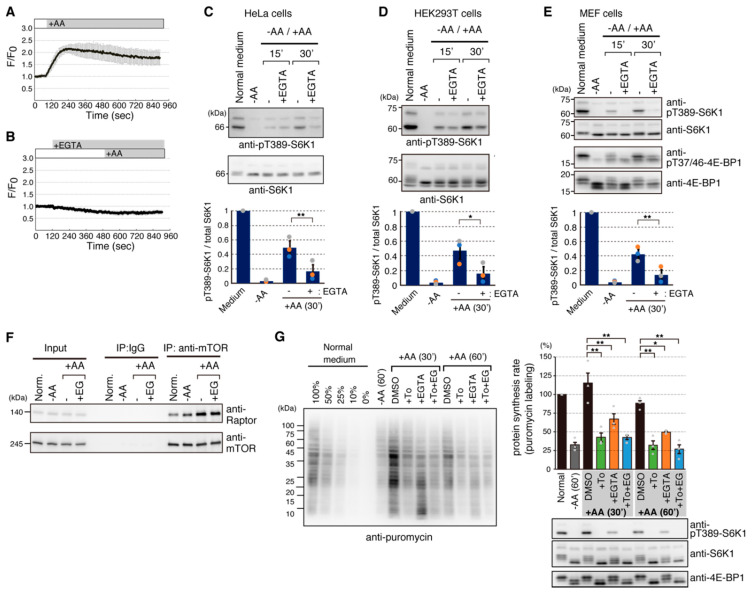
Amino acid-induced Ca^2+^ entry is involved in mTORC1 activation. (**A**,**B**) HeLa cells stably expressing R-GECO1 were deprived of amino acids for 50 min and then an amino acids mixture was added at 120 s. EGTA (final concentration of 3 mM) was added 6 min before the addition of amino acids in (**B**). Ratios of fluorescence signals (F) for R-GECO1 to the average signals before the addition of amino acids (F_0_) are shown. Graphs are shown as means ± SEM (*n* = 20). (**C**–**E**) HeLa (**C**), HEK293T (**D**), and MEF (**E**) cells were cultured in a normal medium or transferred to HBSS for 50 min in (**C**) or 60 min (**D**,**E**) (−AA), and then an amino acid mixture was added for the indicated times. EGTA [final concentration of 3 mM (**C**) or 4 mM (**D**,**E**)] was added 5 min before the addition of amino acids. Cell lysates were analyzed by Western blotting with the indicated antibodies. Graphs represent mean ± SEM of three independent experiments, in which the relative intensity of phospho-T389-S6K1 to total S6K1 in normal medium was set to 1. One-way ANOVA with Tukey’s test, ** *p* < 0.01, * *p* < 0.05. (**F**) HEK293T cells were treated as in (**D**), and cell lysates were subjected to immunoprecipitation (IP) with an anti-mTOR (N-19) antibody. Immuno-complexes were analyzed by Western blotting with anti-mTOR (7C10) and anti-Raptor (10E10) antibodies. (**G**) MEF cells were cultured in a normal medium and transferred to HBSS for 60 min (−AA), and then HBSS was changed to DMEM without FBS [DMEM(−)] for 30 or 60 min (+AA). Pretreatments with Torin1 (+To), EGTA (+EGTA), and both Torin1 and EGTA (+To+EG) were done 5 min before stimulation with DMEM(−). These compounds were also added to DMEM(−) used for stimulation during experiments. Puromycin (10 μg/mL) was added at the end of each condition and incubated for 15 min. (**Left**) Cell lysates were analyzed by Western blotting with an anti-puromycin (12D10) antibody. Cell lysates derived from the normal medium condition corresponding to 0%, 10%, 25%, 50%, and 100% were run for making standard curves. Representative results are shown. (**Right**) Protein synthesis rates were quantified by measuring signals derived from the anti-puromycin antibody as described in Section 4. Data are presented as relative protein synthesis rate with the puromycin-labeled signal from a normal culture medium set to 100. Data are shown as means ± SEM of three independent experiments. One-way ANOVA with Tukey’s test, * *p* < 0.05, ** *p* < 0.01. Representative results of Western blotting with indicated antibodies in the puromycin-labeling experiments are shown below.

Previous studies including our studies demonstrated that addition of amino acids induces a rise in intracellular Ca^2+^ level [3,15,20,21]. To investigate the significance of Ca^2+^ rise in mTORC1 signaling, we established HeLa cells stably expressing R-GECO1 [22], a red fluorescent protein-based Ca^2+^ indicator. As previously reported [15,21], addition of amino acids to amino acid-starved HeLa cells immediately caused intracellular Ca^2+^ rise (Figure 1A). To examine the source of Ca^2+^ leading to a rise in intracellular Ca^2+^ by addition of amino acids, EGTA, a cell-impermeable Ca^2+^ chelator, was added to an extracellular medium to remove free Ca^2+^ from the medium. As shown in Figure 1B, pretreatment of the cells with EGTA effectively prevented the Ca^2+^ rise by addition of amino acids. To exclude the possibility that EGTA had adverse effects on cells, we also used a medium without the addition of Ca^2+^ and found that Ca^2+^ rise was also prevented in this medium (data not shown). These results suggest that the addition of amino acids causes intracellular Ca^2+^ rise by promoting Ca^2+^ entry from the extracellular medium.

Next, we examined whether intracellular Ca^2+^ rise affects amino acid-mediated mTORC1 signaling. As shown in Figure 1C,D, pretreatment of HeLa cells and HEK293T cells with EGTA consistently reduced the phosphorylation of Thr389 on S6K1, a readout of mTORC1 activity, suggesting that mTORC1 activity can be modulated by amino acid-induced Ca^2+^ rise, although the effects of EGTA were slightly different among cell lines. We also tested mouse embryonic fibroblast (MEF) cells and found that Ca^2+^ chelation by EGTA dramatically prevented mTORC1 activation by amino acids as shown by decreased phosphorylation of both S6K1 and 4E-BP1 (Figure 1E). Pretreatment with EGTA unlikely affected the complex integrity of mTORC1 (Figure 1F). Thus, amino acid-induced Ca^2+^ entry appears to be generally important for proper mTORC1 activation.

Since one of the cellular functions of mTORC1 is promotion of protein synthesis by phosphorylating downstream substrates, S6K1 and 4E-BP1, we next investigated whether Ca^2+^ rise also contributed to mTORC1-regulated protein synthesis. To this end, we utilized the puromycin-labeling method, called SUnSET [23,24], to monitor the protein synthesis rate in vivo. For these experiments, we used MEF cells due to the higher dependency of Ca^2+^ chelation (Figure 1E). As shown in Figure 1G, under amino acid-starved conditions, the protein synthesis rate in MEF cells was decreased to about 30% of that in normal medium conditions. Readdition of amino acids for 30 or 60 min resulted in recovery of the protein synthesis rate to that in normal medium conditions. Importantly, pretreatment with Torin1, a potent mTOR inhibitor, prevented the amino acid-induced increase of the protein synthesis rate, indicating that amino acid-induced protein synthesis is mostly mediated through mTOR. Pretreatment with EGTA also reduced amino acid-induced protein synthesis. Moreover, protein synthesis upon pretreatment with both Torin1 and EGTA was similar to that with Torin1 alone, indicating that the inhibitory effect of EGTA on amino acid-induced protein synthesis is caused through specific inhibition of the mTORC1 pathway rather than through inhibition of other pathways. Protein synthesis rate appears to correlate with phosphorylation status of both S6K1 and 4E-BP1 (Figure 1G, right lower panels). Taken together, these results suggest that the intracellular Ca^2+^ rise induced by addition of amino acids plays an important role in the mTORC1 pathway and thereby mTORC1-regulated cellular processes.

### 2.2. Arginine and Lysine Are Largely Responsible for Ca^2+^ Entry

**Figure 2:**
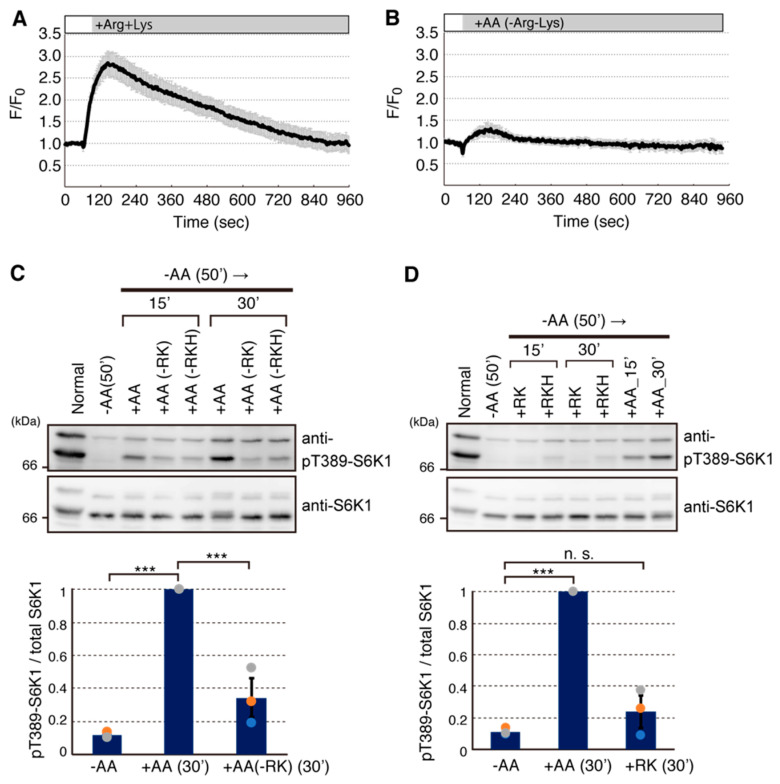
Arginine and Lysine mainly stimulate Ca^2+^ entry. (**A**,**B**) HeLa cells stably expressing R-GECO1 were deprived of amino acids for 50 min, then arginine and lysine (+Arg+Lys) (**A**) or amino acids mixture without arginine and lysine (AA−Arg−Lys) (**B**) were added at 60 s. Ratio of fluorescence signals (F) for R-GECO1 to the average signals before amino acids stimulation (F_0_) were shown. Graphs are shown as mean ± SEM (*n* = 20). (**C**) HeLa cells were cultured in normal medium (Normal) or transferred to HBSS for 50 min (−AA), and then amino acids mixture was added for indicated times. Cells were stimulated with amino acids mixture lacking arginine and lysine (−RK) or arginine, lysine and histidine (−RKH). (**D**) HeLa cells were treated as in (**C**), and amino acids mixture (+AA), arginine and lysine alone (+RK), or arginine, lysine and histidine (+RKH) were added for indicated times. Cell lysates were analyzed by Western blotting with indicated antibodies. (**C**,**D**) Graphs represent mean ± SEM of three independent experiments, in which the relative signal intensity of phospho-T389-S6K1 to total S6K1 in amino acid stimulation (+AA) was set to 1. One-way ANOVA with Tukey’s test, *** *p* <0.001, n.s.; not significant.

To examine which amino acid(s) is responsible for induction of intracellular Ca^2+^ rise, we tested the effect of a set of amino acids on Ca^2+^ rise. Of 13 amino acids tested (Arg, Cystine, Gln, His, Ile, Leu, Lys, Met, Phe, Thr, Trp, Tyr, and Val included in the normal culture medium), Arg and Lys potently induced intracellular Ca^2+^ rise (Figure 2A and data not shown). A previous study has suggested that Leu alone potently induced intracellular Ca^2+^ rise [15]. However, a mixture of amino acids lacking both Arg and Lys but still containing Leu only weakly induced intracellular Ca^2+^ rise (Figure 2B). Thus, our results suggest that Arg and Lys mainly contribute to the amino acid-induced Ca^2+^ rise in HeLa cells. To further test the effects of Arg and Lys on the mTORC1 pathway, we added a mixture of amino acid lacking both Arg and Lys to amino acid-starved cells. Compared with the mixture of 13 amino acids, the lack of both Arg and Lys resulted in reduced phosphorylation of S6K1 (Figure 2C), being consistent with the effect of EGTA pretreatment. In contrast, stimulation with only Arg and Lys barely caused phosphorylation of S6K1 (Figure 2D). We also examined the effects of basic amino acids including His. Although the effect of a mixture of amino acids without Arg, Lys, and His was similar to that of amino acids without Arg and Lys (Figure 2C), the addition of Arg, Lys and His slightly increased S6K1 phosphorylation. However, the effect was much weaker than that of the mixture of 13 amino acids (Figure 2D). These results suggested that basic amino acids, especially Arg and Lys, can induce Ca^2+^ entry to cause an intracellular Ca^2+^ rise, but these amino acids alone are insufficient for proper mTORC1 activation. This is consistent with the idea that Leu is a primary amino acid that induces the essential activation step, i.e., lysosomal translocation of mTORC1.

### 2.3. Amino Acid-Mediated Ca^2+^ Rise Does Not Affect Lysosomal Translocation of mTORC1

**Figure 3:**
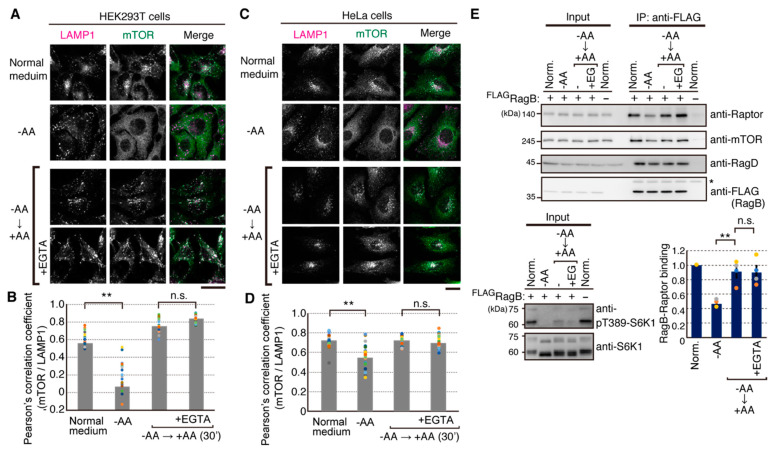
Ca^2+^ entry is not involved in the lysosomal translocation of mTORC1. (**A**,**C**) HEK293T (**A**) and HeLa (**C**) cells were starved for amino acids for 50 min (−AA) and then stimulated with amino acids for 30 min (+AA). EGTA was added 15 min before the addition of amino acids (+EGTA). The cells were then immunostained with anti-mTOR (7C10) and anti-LAMP1 antibodies. Scale bars, 20 μm. (**B**,**D**) Colocalizations of mTOR with LAMP1 were quantified, and Pearson’s correlation coefficient is shown. N = 20. One-way ANOVA with Tukey’s test, ** *p* < 0.01, n.s.; not significant. (**E**) HEK293T cells stably expressing FLAG-tagged RagB (FLAG-RagB) were starved for amino acids for 60 min and then stimulated with amino acids for 30 min. EGTA (final concentration of 4 mM) was added 5 min before stimulation with amino acids for 30 min. Then the cells were treated with a crosslinker DSP for 7 min. Cell lysates (Input) and FLAG immunoprecipitates (IP: anti-FLAG) were analyzed by Western blotting with the indicated antibodies. Asterisk indicates IgG heavy chain. Graphs represent mean ± SEM of four independent experiments, in which relative binding of Raptor to FLAG-RagB in normal medium was set to 1. One-way ANOVA with Tukey’s test, * *p* < 0.05, ** *p* < 0.01, n.s.; not significant.

To examine whether amino acid-induced Ca^2+^ entry affects lysosomal translocation of mTORC1, we next observed subcellular localization of mTOR by immunofluorescence microscopy. As reported previously [18], mTOR signals showed diffused patterns under the amino acid-starved condition in both HeLa and HEK293T cells (Figure 3A–D). Upon addition of amino acids, mTOR signals were colocalized with LAMP1-positive organelles, indicating lysosomal translocation of mTOR (Figure 3A–D). EGTA pretreatment did not prevent the lysosomal translocation in response to amino acids addition in both HeLa and HEK293T cells (Figure 3A–D). As mTORC1 is known to be recruited to the lysosomal surface primarily by binding of the Raptor subunit to heterodimeric Rag GTPases in an amino acid-sensitive manner, we further examined the effect of EGTA pretreatment on amino acid-sensitive binding of RagB and Raptor. As previously demonstrated [18], we established HEK293T cells stably expressing FLAG-RagB, and used in vivo crosslinking analysis for monitoring the binding of FLAG-RagB with Raptor, mTOR, and RagD in response to amino acids. As shown in Figure 3E, amino acid starvation reduced the binding of Raptor and to a lesser extent mTOR to FLAG-RagB. Readdition of amino acids resulted in recovery of the interaction between FLAG-RagB and Raptor to a level similar to that under the normal growth condition. Consistent with the results of immunofluorescence analysis of the lysosomal translocation of mTOR (Figure 3A–D), pretreatment with EGTA did not prevent amino acid-induced interaction of FLAG-RagB with Raptor or mTOR. The amount of RagD binding to FLAG-RagB was not significantly changed during these treatments. The results suggest that the amino acid-induced Ca^2+^ rise does not affect the amino acid-mediated recruitment of mTORC1 onto the lysosomal surface.

### 2.4. Calmodulin (CaM) Is Involved in mTORC1 Activation

**Figure 4:**
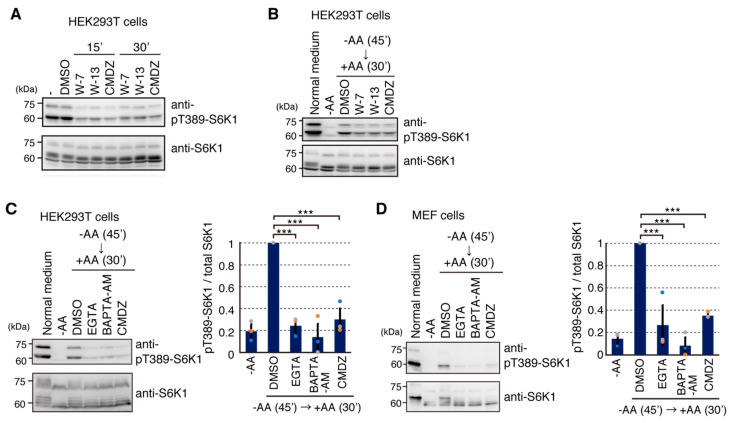
CaM inhibitors generally inhibit mTORC1 activation. (**A**) HEK293T cells were treated with different CaM inhibitors (W-7 (30 μM), W-13 (50 μM) and calmidazolium (CMDZ, 30 μM)) for the indicated times. Cell lysates were analyzed by Western blotting with the indicated antibodies. (**B**) HEK293T cells were transferred to HBSS for 45 min (−AA) and pretreated with the indicated CaM inhibitors for the last 15 min and then stimulated with an amino acid mixture (+AA) for 30 min. Cell lysates were analyzed by Western blotting with the indicated antibodies. (**C**,**D**) HEK293T cells (**C**) or MEF (**D**) cells were transferred to HBSS for 45 min (−AA) and pretreated with the indicated compounds (4 mM EGTA, 50 μM (**C**) or 10 μM (**D**) BAPTA-AM, and 30 μM CMDZ) for the last 5 min and then stimulated with an amino acid mixture (+AA) for 30 min. DMSO was used as a control vehicle for CMDZ and BAPTA-AM. Cell lysates were analyzed by Western blotting with the indicated antibodies. Graphs represent mean ± SEM of three independent experiments, in which relative intensity of phospho-T389-S6K1 to total S6K1 in the amino acid stimulation (DMSO) was set to 1. One-way ANOVA with Tukey’s test, *** *p* < 0.001.

To investigate a key mediator linking Ca^2+^ signals to mTORC1 activation, we first tested the effect of ALG-2 [21,25], because it was reported to be a Ca^2+^-binding protein involved in lysosomal Ca^2+^ release [26]. However, we failed to detect any consistent effects of ALG-2 knockdown on mTORC1 signaling in response to amino acids (data not shown). Previous studies suggested that calmodulin (CaM) can affect mTORC1 signaling [15,27,28]. Therefore, we tested the effect of several CaM inhibitors on mTORC1 signaling. Treatments with three different CaM inhibitors, W-7, W-13 and calmidazolium (CMDZ), consistently reduced the phosphorylation of S6K1 in both normal culture (Figure 4A) and amino acid-starved/amino acids readdition conditions (Figure 4B). In addition, suppression of mTORC1 activity by CMDZ treatment was comparable to that by EGTA and BAPTA-AM in both HEK293T and MEF cells (Figure 4C,D). Thus, CaM appears to be involved in regulation of the mTORC1 pathway both in the basal condition and in response to amino acids.

### 2.5. Ca^2+^/CaM Binds to TSC2

**Figure 5:**
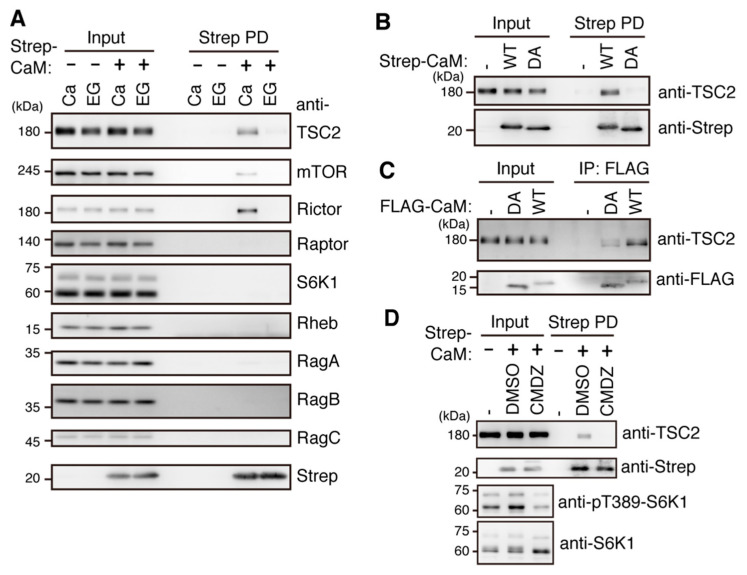
CaM mainly interacts with TSC2 in a Ca^2+^-dependent manner. (**A**) HEK293T cells were transiently transfected with an empty vector or the expression plasmid for Strep-CaM for 24 h. Cell lysates were subjected to pulldown (PD) with Strep beads in the presence of 100 μM CaCl_2_ (Ca) or 5 mM EGTA (EG). Cell lysates (Input) and pulldown products (Strep PD) were analyzed by Western blotting with the indicated antibodies. Representative results of three independent experiments are shown. (**B**) HEK293T cells were transiently transfected with the expression plasmid for Strep-CaM (WT) or Strep-CaM^D21A, D57A, D94A, D130A^, a Ca^2+^ binding defective mutant of CaM (DA), for 24 h. Cell lysates were analyzed as in (**A**) in the presence of 100 μM CaCl_2_. Representative results of two independent experiments are shown. (**C**) HEK293T cells were transiently transfected with the expression plasmid for FLAG-CaM (WT) or FLAG-CaM^D21A, D57A, D94A, D130A^, a Ca^2+^ binding defective mutant of CaM (DA), for 24 h. Cell lysates were immunoprecipitated with the anti-FLAG antibody. IP products and cell lysates were analyzed by Western blotting with the indicated antibodies. (**D**) HEK293T cells were transiently transfected with an empty vector or the expression plasmid for Strep-CaM for 24 h. The cells were left (-) or treated with a vehicle (DMSO) or CMDZ (30 μM) for 60 min. Cell lysates were subjected to pulldown and analyzed by Western blotting with anti-TSC2 (C-20) and anti-Strep antibodies. Representative results of three independent experiments are shown.

To identify potential factors connecting CaM with mTORC1 signaling, we transiently overexpressed Strep-tagged CaM (Strep-CaM) and screened proteins in the presence or absence of Ca^2+^. Among the factors in mTOR signaling tested (mTOR, Raptor, Rictor, TSC2, Rheb, RagA, RagB, RagC, and S6K1), we found that TSC2 was bound to Strep-CaM in a Ca^2+^-dependent manner (Figure 5A), consistent with the previous result using CaM sepharose [29]. Although mTOR and Raptor were previously reported to bind to CaM sepharose beads as well [27], our results indicated that the interaction of CaM with mTOR was weaker than that with TSC2 and that CaM binding to Raptor was below the detectable level. Surprisingly, we found that Rictor, an mTORC2 component, could bind to CaM more strongly in a Ca^2+^-dependent manner. These results suggest that the weak association of mTOR with CaM might reflect indirect binding through Rictor subunit. Thus, TSC2 might be a strong candidate linking Ca^2+^ signaling to mTORC1 regulation. To further define the binding mode between CaM and TSC2, we used a Ca^2+^ binding defective mutant of CaM [30] (Strep-CaM^DA^, in which four Ca^2+^ coordinate Asp residues were changed to Ala) and found that Strep-CaM^DA^ reduced binding to TSC2 (Figure 5B) and that FLAG-tagged CaM and CaM^DA^ behaved similar to Strep-CaM (Figure 5C). We further tested whether CaM-TSC2 interaction was sensitive to a CaM inhibitor. Indeed, CMDZ treatment, which decreased phosphorylation of Thr389 of S6K1, also reduced binding of CaM to TSC2 (Figure 5D).

**Figure 6:**
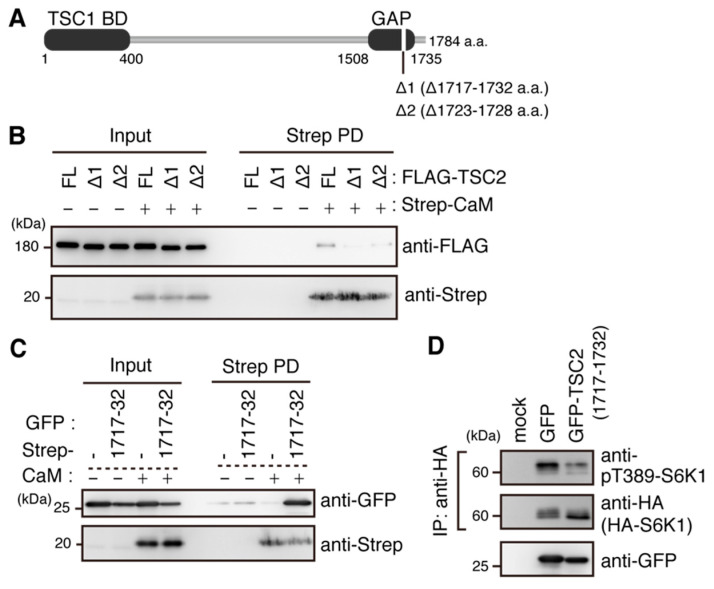
CaM interacts with TSC2 at its GAP domain. (**A**) Schematic representation of the TSC2 structure. TSC1 BD: TSC1 binding domain. GAP: GAP domain. Δ1 and Δ2 represent a TSC2 mutant lacking a predicted CaM binding region (1717–1732 a.a.) and a patient-derived TSC2 mutant lacking 1723–1728 a.a., respectively. (**B**) HEK293T cells were transiently transfected with expression plasmids for FLAG-TSC2 (FL), FLAG-TSC2Δ1717–1732 (Δ1), and FLAG-TSC2Δ1723–1728 (Δ2) together with the plasmid for Strep-CaM or an empty vector for 24 h. Cell lysates were subjected to pulldown and analyzed by Western blotting with anti-FLAG and anti-Strep antibodies. Representative results of three independent experiments are shown. (**C**) HEK293T cells were transiently transfected with expression plasmids for GFP and GFP-TSC2 (1717–1732) together with the plasmid for Strep-CaM or an empty vector for 24 h. Cell lysates were subjected to pulldown and analyzed by Western blotting with anti-GFP and anti-Strep antibodies. (**D**) HEK293T cells were transiently transfected with expression plasmids for GFP and GFP-TSC2 (1717–1732) together with the expression plasmid for HA-S6K1 for 24 h. Cell lysates were subjected to immunoprecipitation (IP) with an anti-HA (3F10) antibody. IP products and cell lysates were analyzed by Western blotting with the indicated antibodies. Representative results of two independent experiments are shown.

In a previous study, a CaM-binding site was predicted in the C-terminal region of TSC2 [29]. To examine whether the predicted CaM-binding site within TSC2 contributes to the interaction with CaM, a TSC2 mutant lacking the region (TSC2Δ1717–1732) was tested (Figure 6A, Δ1). Note that the amino acid numbering of TSC2 is based on isoform 4 of human TSC2 in Uniprot throughout this study. Compared with full-length TSC2 (FL), interaction between FLAG-TSC2Δ1717–1732 and Strep-CaM was dramatically decreased (Figure 6B, Δ1). Furthermore, the region of TSC2 corresponding to 1717–1732 a.a. was sufficient for binding of Strep-CaM (Figure 6C). Within the identified CaM-binding region, a naturally occurring TSC2 mutant lacking 6 a.a. (HIKRLR) in TSC patients showing epilepsy has been identified [31] (Figure 6A, Δ2). We also tested binding of CaM to TSC2 lacking the 6 a.a. (TSC2Δ1723–1728) and found that the TSC2 mutation also reduced binding to CaM (Figure 6B, Δ2), suggesting that loss of CaM binding to the TSC2 mutation might be associated with onset of TSC diseases. Consistent with both reduction of phosphorylated S6K1 and reduced interaction between CaM and TSC2 by a CaM inhibitor (Figure 5D), overexpression of the minimal binding region of TSC2 with CaM (TSC2 1717–1732 a.a.) resulted in decrease in the phosphorylated form of S6K1 (Figure 6D). Thus, it is likely that binding of Ca^2+^/CaM with TSC2 underlies the mechanistic basis for Ca^2+^ signaling to modulate mTORC1 activity.

**Figure 7:**
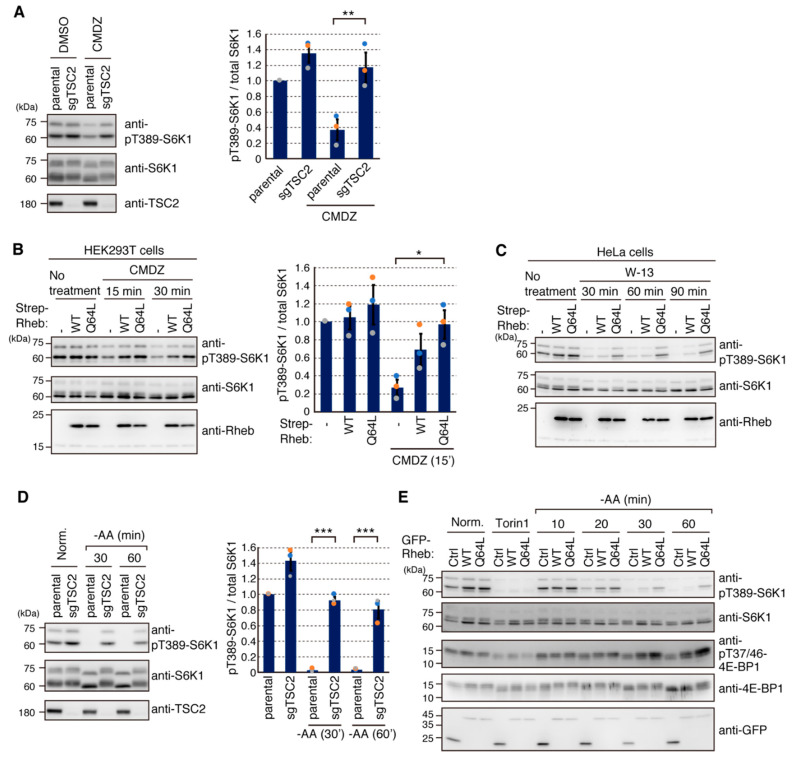
Inhibitory effect of CaM inhibitors on mTORC1 is exerted through inactivation of the TSC2-Rheb axis. (**A**) HEK293T (parental) and HEK293T TSC2 KO (sgTSC2) cells were treated with DMSO or CMDZ (30 μM) for 30 min. Cell lysates were analyzed by Western blotting with the indicated antibodies. Graphs represent mean ± SEM of three independent experiments, in which relative intensity of phospho-T389-S6K1 to total S6K1 in the parental cells (DMSO) was set to 1. One-way ANOVA with Tukey’s test, ** *p* < 0.01. (**B**) HEK293T cells stably expressing Strep-Rheb (WT) or high GTP-loaded mutant Strep-Rheb^Q64L^ (Q64L) were treated with CMDZ (30 μM) for the indicated times, and cell lysates were analyzed as in (**A**). Graphs represent mean ± SEM of three independent experiments, in which relative intensity of phospho-T389-S6K1 to total S6K1 in the control cells was set to 1. One-way ANOVA with Tukey’s test, * *p* < 0.05. (**C**) HeLa cells stably expressing Strep-Rheb (WT) or high GTP-loaded mutant Strep-Rheb^Q64L^ (Q64L) were treated with W-13 (50 μM) for the indicated times, and cell lysates were analyzed as in (**A**). (**D**) HEK293T (parental) and HEK293T TSC2 KO (sgTSC2) cells were cultured in the normal condition (Norm.) or starved with amino acids (−AA) for the indicated times, and cell lysates were analyzed as in (**A**). Graphs represent mean ± SEM of three independent experiments, in which relative intensity of phospho-T389-S6K1 to total S6K1 in the normal medium in parental cells was set to 1. One-way ANOVA with Tukey’s test, *** *p* < 0.001. (**E**) HEK293T cells stably expressing GFP (Ctrl), GFP-Rheb (WT) or high GTP-loaded mutant GFP-Rheb^Q64L^ (Q64L) were treated as in (**D**). Torin1 (250 nM) was added to the normal medium for 20 min. Cell lysates were analyzed as in (**A**).

To further confirm that TSC2 is the target of CaM involved in regulation of the mTORC1 pathway, we investigated the inhibitory effect of CaM inhibitors on mTORC1 activity in HEK293T TSC2 KO cells. Consistent with identification of TSC2 as a target of CaM in mTORC1 signaling (Figure 5 and Figure 6), the decrease in phosphorylation of S6K1 by CMDZ treatment was dramatically prevented in TSC2 KO cells compared to that in parental cells (Figure 7A). To further examine whether the inhibitory effects of CaM inhibitors on mTORC1 activity can be rescued by the increase of GTP-bound Rheb, we made cells stably expressing Strep-Rheb and Strep-Rheb^Q64L^. As shown in Figure 7B,C, although Strep-Rheb showed only weak resistance to CaM inhibitors (CMDZ or W-13 treatment), higher levels of GTP-bound Rheb (Strep-Rheb^Q64L^) showed much greater resistance to the CaM inhibitor-mediated decrease in phosphorylated S6K1. These results indicate that CaM inhibitors likely decreased GTP-loading of Rheb, leading to reduction of S6K1 phosphorylation. Taken together, our results suggest that Ca^2+^/CaM binding to TSC2 likely results in the inhibition of TSC2 action toward Rheb GTPase, which ultimately leads to an increase of mTORC1 activity. To confirm that TSC2 is indeed involved in mTORC1 regulation in response to amino acid availability, TSC2 KO cells were tested for amino acid starvation as well. As shown in Figure 7D, TSC2 KO cells exhibited strong resistance to dephosphorylation of S6K1 in response to amino acid deprivation. In addition, cells expressing active Rheb (Rheb^Q64L^) were resistant to amino acid deprivation-induced mTORC1 inactivation (Figure 7E). The remaining phosphorylation of S6K1 and 4E-BP1 indeed reflected high mTORC1 activity, since treatment with Torin1 dramatically reduced phosphorylation of S6K1 and 4E-BP1 even in these cells. Thus, the decrease of GTP-bound Rheb pools by action of TSC2 appears to be prerequisite for inactivation of mTORC1 during amino acid deprivation. These results indicate that amino acid availability activates mTORC1 not only via regulation of Rag GTPases but also via regulation of TSC2-Rheb GTPase.

## 3. Discussion

**Figure 8:**
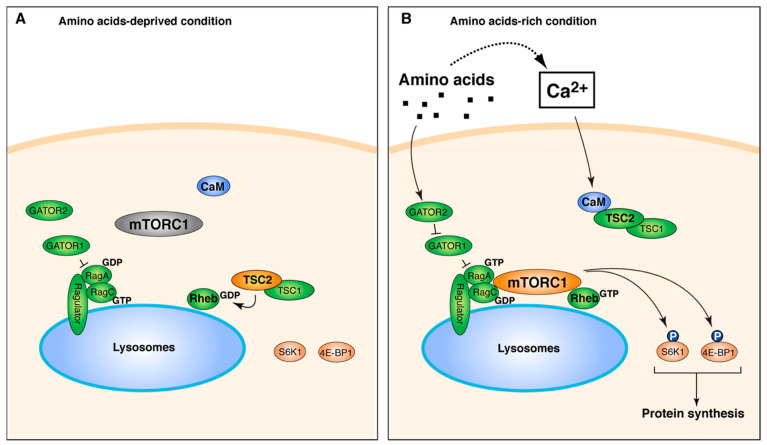
A model for mTORC1 regulation by Ca^2+^/CaM-mediated regulation via TSC2-Rheb axis. (**A**) Under the condition deprived of amino acids, heterodimeric Rag GTPases (RagA and RagC are shown) are inactive, and mTORC1 is diffusely localized in the cytoplasm. TSC2 is recruited to lysosomes and acts as GAP to inactivate Rheb GTPase. (**B**) Under the amino acids-rich condition, amino acids regulate GATOR complexes (GATOR1 and GATOR2) to activate Rag GTPases, which in turn recruit mTORC1 at the lysosomal surface. Simultaneously, amino acids also induce Ca^2+^ entry. The elevation of intracellular Ca^2+^ levels can be sensed by CaM. Ca^2+^/CaM binds to TSC2, which likely inactivates TSC2, and leads to activation of Rheb GTPase and thereby of mTORC1.

In the present study, we found a novel pathway leading to mTORC1 activation, in which Ca^2+^-mediated signaling converges on TSC2 via CaM. The addition of amino acids stimulates Ca^2+^ entry, which can be sensed by CaM, and Ca^2+^/CaM is in turn associated with TSC2 to regulate the TSC2-Rheb axis. Thus, our results demonstrate that amino acid availability can regulate mTORC1 kinase activation through TSC2-Rheb GTPase in addition to canonical lysosomal translocation of mTORC1 to properly modulate mTORC1-dependent metabolism in order to meet cellular demands (Figure 8). Our results also suggest that intracellular Ca^2+^ levels are able to contribute to TSC2 action via Ca^2+^/CaM and thereby add multiple layers of regulation to mTORC1 activation.

As Ca^2+^ is a fundamental second messenger linking many physiological conditions to intracellular activity of cells, our results also indicate that intracellular Ca^2+^ status and/or extracellular Ca^2+^ status are responsible for mTORC1 regulation and are therefore extremely important in some physiological and pathological cases. It has been demonstrated that Ca^2+^ signaling is linked to muscle hypertrophy and atrophy [32]. For example, muscle-specific KO of STIM1, an essential activator of store-operated Ca^2+^ entry (SOCE), resulted in reduced muscle growth concomitant with reduced activation of Akt or Erk1/2, indicating that STIM1-mediated regulation of intracellular Ca^2+^ concentration is required for proper muscle hypertrophy [33]. The results of the present study indicate that proper Ca^2+^ elevation during amino acid readdition is required for promotion of protein synthesis. This finding might explain muscle hypertrophy in response to exercise and protein supplementation, because it was reported that increase in Ca^2+^ contributes to muscle hypertrophy [34]. In addition, perturbation of mTORC1 pathway regulation by aberrant Ca^2+^ levels might be one of the key mechanistic bases for onset of aberrant hypertrophy. Indeed, consistent with the results of our study, a very recent study on mice with cardiac hypertrophy showed that higher Ca^2+^ levels induced by aberrant Ca^2+^ entry by overexpression of SARAF, a negative regulator of SOCE, promotes hypertrophy at least in part through mTORC1 hyperactivation [35]. Thus, our findings might provide a basis for the development of a medicine to overcome this pathological hypertrophy. Moreover, our findings might be associated with antitumor functions of CD8^+^ T cells, because it was reported that activation of mTORC1 via Rag GTPase by amino acids [36] and Ca^2+^ entry via modulation of SOCE [37] regulate antitumor immunity.

We find that the addition of amino acids, especially Arg and Lys, promotes Ca^2+^ entry from extracellular medium. However, it remains unknown what kinds of Ca^2+^ channels are involved in these amino acid-induced processes. Multiple different Ca^2+^ channels might be involved in amino acid-dependent Ca^2+^ entry. It is also possible that depolarization might be associated with Ca^2+^ entry because Arg is reported to stimulate Ca^2+^ entry via depolarization in pancreatic β cells [38]. Further studies are necessary for identification of amino acid-induced Ca^2+^ channels.

Previous studies have shown that CaM positively acted on the mTORC1 pathway in two different manners. First, Ca^2+^/CaM was reported to interact with hVps34, a PI3 kinase that produces phosphatidylinositol 3-phosphate (PI3P) [15]. The production of PI3P might recruit phospholipase D1 at the lysosomal surface to activate mTORC1 [39]. Secondly, Ca^2+^/CaM was reported to bind directly to mTORC1, leading to promotion of mTORC1 kinase activity by an unknown mechanism [27]. We cannot rule out the possibility that these two different actions of CaM are also exerted in parallel with Ca^2+^/CaM binding to TSC2 to modulate mTORC1. However, we propose that TSC2 is a major contributor of the Ca^2+^/CaM action toward mTORC1 signaling, since the inhibitory effect of CaM inhibitors was rescued by TSC2 KO or by the GTP-bound form of Rheb (Rheb^Q64L^) (Figure 7). One of the surprising results is strong binding of CaM to Rictor. This result suggested that CaM might affect mTORC2 action. Since mTORC2 phosphorylates and activates Akt, which in turn inactivates TSC2, it is possible that CaM also indirectly affects TSC2 phosphorylation and activity. However, our preliminary results suggested that the phosphorylation status of TSC2 at Ser939 was not altered during EGTA or BAPTA-AM treatment. Therefore, the major action of Ca^2+^/CaM toward mTORC1 signaling is most likely mediated by interaction with TSC2 rather than by a change of mTORC2 and Akt activity.

We demonstrated that deletion of 1717–1732 a.a. in TSC2 resulted in loss of binding with CaM (Figure 6B). Indeed, a previous study suggested that the C-terminal region of TSC2 might contribute to the binding to CaM, although the detailed binding region was not determined in that study [29]. Our data indicated that the 1717–1732 a.a. region of TSC2 is necessary and sufficient for CaM binding (Figure 6B,C). As the region required for binding to CaM is located in the C-terminal portion of the GAP domain, it is possible that CaM binding to the TSC2 GAP domain decreases GAP activity toward Rheb. Consistent with this idea, the loss of CaM binding to TSC2 caused by a CaM inhibitor resulted in decreased mTORC1 activity. Similarly, mTORC1 activity was decreased by overexpression of the minimal binding region of TSC2 (1717–1732). These results are most likely due to the relief of Ca^2+^/CaM-mediated repression of GAP activity of TSC2. Very recently, the cryo-EM structure of the human TSC complex, which is a pentameric protein complex consisting of two TSC2 molecules, two TSC1 molecules and one TBC1D7 molecule, was reported [40]. The CaM binding region of TSC2 (1717–1732) forms a characteristic helix pair with the N-terminal region (1502–1513) of the GAP domain, and the helix pair is thought to support TSC2-Rheb interactions [40]. Although it is not known how CaM binds to the TSC2 (1717–1732) region, binding of Ca^2+^/CaM might affect the structure of the helix pair and thus the overall structure of the catalytic domain of TSC2, leading to inactivation of TSC2. In fact, multiple phosphorylation sites that are known to affect TSC2 GAP catalytic activity are located far from the catalytic domain of TSC2 [11,41,42,43], indicating that catalytic activity of TSC2 can be allosterically controlled by a wide array of modifications, most likely by affecting the overall structure of the GAP catalytic domain.

Our findings suggest that TSC2 activity can be regulated by amino acid availability via Ca^2+^/CaM. Consistently, several studies suggested that TSC2 activity and thereby GTP-loaded Rheb are regulated by amino acid availability. GTP/GDP-bound forms of Rheb were reported to be changed in response to amino acid availability [7,44,45]. Mechanistically, it has been reported that amino acids as well as growth factors are able to regulate the subcellular localization of TSC2 to control Rheb activity. Under the condition of deprivation of amino acids or growth factors, TSC2 is relocalized to the lysosomal surface, where Rheb is believed to reside, and thereby leads to reduction of GTP-bound Rheb to inactivate mTORC1 [6,19]. However, our preliminary results suggest that Ca^2+^/CaM appears not to regulate TSC2 subcellular localization during the change of amino acid availability. Therefore, interaction of Ca^2+^/CaM with TSC2 might primarily affect catalytic activity of TSC2.

Since TSC2 is one of the most important negative regulators of mTORC1 signaling, our results showing Ca^2+^/CaM binding to TSC2 are fascinating for the development of a new compound activating TSC2. As shown in Figure 6D, overexpression of the TSC2 (1717–1732) region resulted in decreased mTORC1 signaling. Thus, new compounds that specifically prevent CaM binding to TSC2 will be promising for the development of a new medicine to control mTORC1 signaling. Future works on the mechanistic basis for CaM-mediated regulation of TSC2 will thus expand our strategies to combat various metabolic diseases and/or cancer progression associated with aberrant mTORC1 signaling.

## 4. Materials and Methods

### 4.1. Antibodies and Reagents

Anti-pThr389-S6K1 (#9205 and #9234), anti-S6K1 (#9202), anti-pThr37/46-4E-BP1 (236B4, #2855), anti-4E-BP1 (53H11, #9644), anti-mTOR (7C10, #2983), and anti-TSC2 (D93F12, #4308) were purchased from Cell Signaling Technology (Danvers, MA, USA). Anti-S6K1 (C-18, sc-230), anti-Raptor (10E10, sc-81537), anti-Rictor (H-11, sc-271081), anti-TSC2 (C-20, sc-893), anti-Myc (9E10, sc-40), and anti-GST (Z-5, sc-459) were from Santa Cruz Biotechnology (Dallas, TX, USA). Anti-HA (3F10, 11867423001), and anti-FLAG (M2, F1804) were from Sigma-Aldrich (St. Louis, MO, USA). Anti-Strep tagII (PAB16603) was from Abnova (Taipei, Taiwan). Anti-puromycin (12D10, MABE343) was from Millipore (Temecula, CA, USA). The anti-LAMP1 (H4A3) antibody was from BD Transduction Laboratories (San Jose, CA, USA). Horseradish peroxidase (HRP)-conjugated goat antibodies against mouse IgG and rabbit IgG and an HRP-conjugated rabbit antibody against goat IgG were from Jackson ImmunoResearch (West Grove, PA, USA). For immunofluorescence, AlexaFluor488, AlexaFluor555, or AlexaFluor647-conjugated donkey anti-mouse IgG, AlexaFluor488, AlexaFluor555, or AlexaFluor647-conjugated donkey anti-rabbit IgG, and AlexaFluor488, AlexaFluor555, or AlexaFluor647-conjugated donkey anti-goat IgG antibodies were purchased from Invitrogen (Carlsbad, CA, USA) and used as secondary antibodies. BAPTA-AM solution (348-05451) was purchased from Wako (Tokyo, Japan). W-7 (14826), W-13 (14277) and Calmidazolium (14442) were from Cayman Chemical (Ann Arbor, MI, USA). Torin1 (T5870) was from LKT laboratories (St. Paul, MN, USA).

### 4.2. Plasmids

The expression plasmid for HA-S6K1 (pRK5-HA-S6K1) was described previously [46]. The expression plasmid for FLAG-TSC2 (pcDNA3 Flag TSC2) was a gift from Brendan Manning (Addgene plasmid # 14129) [11]. Note that this TSC2 plasmid encodes 1784 residues that correspond to isoform 4 under Uniprot, and we used this numbering throughout this paper.

The expression plasmid for Myc-Rheb (pcDNA3.1-Myc-Rheb) was constructed by insertion of a PCR fragment of human Rheb1 amplified from human cDNA using primers (aatggatccatgccgcagtccaagtcccggaag and cccaaatgatatctttcaggttaacagaag) into BamHI and EcoRV sites of pcDNA3.1-Myc-C (a gift from Dr. T. Maeda). The expression plasmid for Myc-Rheb^Q64L^ (pcDNA3.1-Myc-Rheb^Q64L^) was generated by site-directed mutagenesis using primers (agccggtctagatgaatattctatctt and tcatctagaccggctgtgtctacaagt). The expression plasmid for Strep-CaM (pEXPR-IBA105-B-CALM1) was constructed by inserting a PCR fragment of human CALM1 amplified using primers (ccgcgggaattcggggatccatggctgatcagctgaccgaag and cgcaagcttggtacctcgagtcattttgcagtcatcatctgtacg) and cDNA from HeLa cells into BamHI and XhoI sites of pEXPR-IBA105-B (laboratory stock). Note that the amplified CALM1 sequence had many nucleotide differences from the cDNA database but no amino acid substitution. Therefore, we used the amplified CALM1 cDNA for expression of CaM. The expression plasmid for Strep-CaM^D21A, D57A, D94A, D130A^ (pEXPR-IBA105-B-CALM1^D21A, D57A, D94A, D130A^) was created by site-directed mutagenesis using primers (cctattcgcgaaagatggcgatggcacc, ccatctttcgcgaatagggagaaggcttcc, aagtggctgcagatggtaatggcaccatt, accatctgcagccacttcattgatcatat, agtcttcgcgaaggatggcaatggttat, atccttcgcgaagactcggaatgcctca, aggctgcgatcgatggagatggccag, and catcgatcgcagcctccctgatcatct). pcDNA3.1-FLAG-A-CALM1 was generated by inserting a PCR fragment of CALM1 using primers (cgataaggtacctaggatcctcgctgatcagctgaccgaaga and cctctagactcgagcggccgctcattttgcagtcatcatctg) into pcDNA3.1-FLAG-A (a gift from Dr. T. Maeda). Then the expression plasmid for FLAG-CaM (pHUEV-FLAG-CALM1) was constructed by inserting a PmeI-XbaI fragment of pcDNA3.1-FLAG-A-CALM1 into SmaI and XbaI sites of pHEK293 Ultra Expression Vector I (Takara, Shiga, Japan). The expression plasmid for FLAG-CaM^D21A, D57A, D94A, D130A^ (pHUEV-FLAG-CALM1^D21A, D57A, D94A, D130A^) was constructed similarly.

To generate an expression plasmid for FLAG-TSC2^Δ1717–1732^ (pcDNA3-FLAG-TSC2^Δ1717–1732^), a PCR fragment having the corresponding deletion was amplified using primers (gctccaaccccaccgatatctacccttcgaaggaggaagccgcctactcc and atagaatagggccctctagaactagtggatc) and inserted into EcoRV and XbaI sites. The expression plasmid for a patient-derived mutation of TSC2^Δ1723–1728^ (pcDNA3-FLAG-TSC2^Δ1723–1728^) was constructed similarly using a PCR fragment with primers (ctccaaccccaccgatatctacccctccaagtggattgcccggctccgtcaacggatctgcgaggaagcc and atagaatagggccctctagaactagtggatc).

To generate the expression plasmid for GFP-TSC2 (1717–1732 a.a.), oligonucleotides (tcgaggatggattgcccggctccgccacatcaagcggctccgccagcggatctgctag and gatcctagcagatccgctggcggagccgcttgatgtggcggagccgggcaatccatcc) were annealed and ligated into XhoI and BamHI sites in pSGFP2-C1 [47].

For PCR cloning, PrimeSTAR Max DNA polymerase (Takara) was used for amplification, except for cloning human Rheb1 cDNA, which was amplified with Pyrobest DNA polymerase (Takara). pCX4pur-R-GECO1 was a laboratory stock.

pEXPR-IBA105-B-Rheb was generated by inserting a BamHI-XhoI fragment of pcDNA3.1-Myc-Rheb into BamHI and XhoI sites of pEXPR-IBA105-B. pCX4pur-Strep-tagII-Rheb was constructed by using primers (tcctctagactgccggatccgccaccatggctagctggagcc and cacgcgtcggtccggaattctcacatcaccgagcatgaag) and pEXPR-IBA105-B-Rheb as a template. pCX4pur-Strep-tagII-Rheb^Q64L^ was constructed similarly.

pRK5-FLAG-RagB was constructed by inserting annealed primers (cgatgccaccatggactacaaggatgacgatgacaagggtggtggtggtggtgcg and tcgacgcaccaccaccaccacccttgtcatcgtcatccttgtagtccatggtggcat) into SalI and ClaI sites of pRK5-HA-GST-RagB. pCX4pur-FLAG-RagB was generated by inserting a ClaI-NotI fragment of pRK5-FLAG-RagB into BamHI and NotI sites of pCX4pur.

eSpCas9(1.1)-2A-Puro-TSC2-sg2 was constructed by inserting annealed oligos (caccggacggagtttatcatcaccg and aaaccggtgatgataaactccgtcc) into the BbsI site of eSpCas9(1.1)-2A-Puro [21]. All sequences of constructed plasmids were verified by DNA sequencing.

### 4.3. Mammalian Cell Culture, Transfection and Retroviral Infection

HeLa, MEF, and HEK293T cells were grown at 37 °C in Dulbecco’s modified Eagle’s medium (DMEM) with 1000 mg/L glucose (Nissui, Tokyo, Japan) or with 4500 mg/L glucose (for HEK293T cells) supplemented with 5% fetal bovine serum under a 5% CO_2_ atmosphere. Transient transfection of the indicated plasmids was performed using Polyethylenimine “Max” (PEImax) (#24765, Polysciences Inc., Warrington, PA, USA) for HEK293T cells and FuGENE6 (Promega, Madison, WI, USA) for HeLa cells according to the manufacturer’s instructions. HeLa cells stably expressing R-GECO1 and HEK293T cells stably expressing FLAG-RagB, Strep-Rheb, or Strep-Rheb^Q64L^ were generated by retroviral infection and selected with 1 μg/mL puromycin as essentially described previously [21]. Retroviruses were prepared from culture media of PLAT-A cells (kindly provided by Dr. Toshio Kitamura, The University of Tokyo) that had been transfected with pCX4pur-R-GECO1, pCX4pur-FLAG-RagB, pCX4pur-Strep-tagII-Rheb, pCX4pur-Strep-tagII-Rheb^Q64L^, pCX4pur-SGFP2-Rheb, or pCX4pur-SGFP2-Rheb^Q64L^ using FuGENE6.

Amino acid starvation and readdition were done as described previously [48] with modifications. In brief, cells were washed once with Hank’s balanced salt solution (HBSS) and incubated in HBSS for 50–60 min. For replenishment of amino acids, an amino acid mixture consisting of MEM Amino Acids solution (M5550, Sigma-Aldrich, St. Louis, MO, USA) and glutamine was added at the following final concentrations (mg/L): L-Arg, 84; L-Cys, 48; L-His, 84; L-Ile, 105; L-Leu, 105; L-Lys, 145; L-Met, 30; L-Phe, 66; L-Thr, 95; L-Trp, 20; L-Tyr, 72; L-Val, 94; L-Gln, 584. In Figure 1G, DMEM without FBS was used for amino acid readdition.

### 4.4. Generation of TSC2 Knockout HEK293T Cells

Generation of TSC2 knockout HEK293T cells by the CRISPR/Cas9 system was done as described previously [21]. Briefly, HEK293T cells were transfected with the plasmid [eSpCas9(1.1)-2A-Puro-TSC2-sg2] using PEImax. After 24 h, the cells were plated on 96-well plates, and single colonies were selected. TSC2 knockout cells were identified by Western blotting with two different anti-TSC2 antibodies.

### 4.5. Analysis of Protein Synthesis Rate

The Surface Sensing of Translation (SUnSET) method was used for monitoring protein synthesis rate. After treatments with various conditions shown in Figure 1G, MEF cells were finally treated with 10 μg/mL puromycin for 15 min to label nascent polypeptides with puromycin at their C-terminus. Then the cells were washed once with ice-cold PBS(−) and they were lysed with 1x sample buffer (62.5 mM Tris-HCl (pH 6.8), 2% SDS, 10% glycerol, 0.01% BPB, 50 mM NaF) supplemented with protease inhibitors (3 μg/mL Leupeptin, 0.1 mM Pefabloc, 1 μM Pepstatin A) and boiled for 5 min. Cell lysates were collected after centrifugation at 14,000 rpm for 2 min. Protein concentrations of the cell lysates were adjusted by measuring protein concentration using a BCA protein assay kit (ThermoFisher Scientific, Waltham, MA, USA). After addition of DTT at the final concentration of 100 mM, the cell lysates were boiled for 5 min. Puromycin-labeled proteins were analyzed by Western blotting using the anti-puromycin (12D10) antibody. Signal intensities were measured using Image J (NIH, Bethesda, MD, USA). Puromycin-labeled signals from different conditions were calculated on the basis of a standard curve using signal intensities from 0%, 10%, 25%, 50%, and 100% of cell lysates derived from the normal medium condition. To avoid signals from the next lanes, we ran samples from weaker signal to stronger signal in order for the purpose of quantification.

### 4.6. Live-Cell Imaging

Live-cell imaging analyses were performed essentially as described previously [21]. Briefly, HeLa cells stably expressing R-GECO1 were seeded in a glass-bottom dish (Asahi Glass, Tokyo, Japan) and treated as follows. For amino acid starvation, the medium was replaced with HBSS for 60 min. Time-lapse images were acquired under an FV1000-D confocal laser-scanning microscope equipped with a 1.35 numerical aperture oil-immersion objective (UPLSAPO60XO, Olympus, Tokyo, Japan) before and after addition of an amino acid mixture at 37 °C. For EGTA pretreatment, EGTA was added at the final concentration of 4 mM.

### 4.7. Indirect Immunofluorescence

HeLa and HEK293T cells were seeded onto cover glasses coated with poly L-Lysine. After 24 h, the cells were washed once with PBS(−) and fixed with 4% PFA/PBS(−) for 15 min, followed by washing three times with PBS(−). Then the cells were permeabilized with 0.05% Triton X-100/PBS(−) for 5 min, followed by washing three times with PBS(−). After blocking with 0.25% BSA/PBS(−) for 60 min, the cells were incubated with primary antibodies at 4 °C overnight and washed five times with 0.25% BSA/PBS(−). The cells were then incubated with secondary antibodies at room temperature for 60 min in the dark and then washed five times with 0.25% BSA/PBS(−) and mounted. Images were acquired under an FV1000-D confocal laser-scanning microscope.

### 4.8. Colocalization Analysis

Colocalization (Pearson’s correlation coefficient) of mTOR with LAMP1 was calculated using ImageJ Coloc 2 Plugin. After background subtraction using Sliding paraboloid and Disable smoothing settings, individual cells were manually selected and calculated.

### 4.9. Cell Lysate Preparation, Pulldown and Western Blotting

Cells were washed once with ice-cold PBS(−) and lysed with lysis buffer TX (50 mM Tris-HCl (pH 7.5), 150 mM NaCl, 1% Triton X-100, 50 mM NaF, 10 mM β-glycerophosphate) containing 3 μg/mL leupeptin, 1 μM pepstatin A, and 0.1 mM pefabloc and centrifuged at 9000× *g* for 10 min to obtain cell lysates.

In Figure 1F, HEK293T cells grown on a 100-mm dish were washed once with ice-cold PBS(−) and lysed with 1 mL of lysis buffer A (40 mM HEPES-NaOH (pH 7.4), 120 mM NaCl, 0.3% CHAPS, 50 mM NaF) containing 9 μg/mL leupeptin, 3 μM pepstatin A, and 0.3 mM pefabloc. After centrifugation at 9000× *g* for 10 min, the supernatant was incubated overnight with anti-mTOR (N-19). The immunocomplexes were collected by the addition of Dynabeads protein G (Invitrogen) and washed three times with lysis buffer A.

In Figure 5A,B, HEK293T cells were lysed with lysis buffer B (50 mM Tris-HCl (pH 7.5), 150 mM NaCl, 0.3% CHAPS, 10 mM β-glycerophosphate) containing 3 μg/mL leupeptin, 1 μM pepstatin A, and 0.1 mM pefabloc and centrifuged at 9000× *g* for 10 min. The cell lysates were incubated overnight with MagStrep “type 3” (IBA, 2-4090-002, Göttingen, Germany) in the presence of 100 μM CaCl_2_ or 5 mM EGTA and washed three times as above. In Figure 5C, anti-FLAG M2 Agarose Affinity Gel (A2220, Sigma) was used instead of MagStrep “type 3” and the experiment was done similarly.

In Figure 1G and Figure 4C,D, for clear separation of phosphorylated S6K1 species, acrylamide solutions with low percentage of bisacrylamide (acrylamide:bisacrylamide = 30:0.225) was used instead of our normal acrylamide solution (acrylamide:bisacrylamide = 30:0.8).

### 4.10. In Vivo Crosslinking

Crosslinking was done essentially as described previously [18]. DSP was dissolved in DMSO to a final concentration of 250 mg/mL to make a 250X stock solution for the in vivo cross-linking. HEK293T cells and HEK293T cells stably expressing FLAG-RagB that were grown on 100-mm dishes were starved for amino acids and then stimulated as described above, and EGTA was added to a final concentration of 4 mM for EGTA pretreatment. After starvation and stimulation, the cells were rinsed once with PBS(−) and incubated with 4 mL of 1 mg/mL DSP/PBS for 7 min at room temperature. Then, for cross-linking reaction quenching, 1.5 M Tris-HCl (pH 7.4) was added to a final concentration of 100 mM and incubated for 1 min. The cells were rinsed once with ice-cold PBS(−) and lysed with 1 mL of RIPA buffer (40 mM HEPES-NaOH (pH 7.4), 2 mM EDTA, 10 mM β-glycerophosphate, 1% sodium deoxycholate, 1% NP-40, 0.1% SDS) containing 3 µg/mL leupeptin, 1 μM pepstatin A, and 0.1 mM pefabloc and centrifuged at 9000× *g* for 10 min. The cells lysates were incubated with FLAG M2 agarose beads for 3 h with rotation at 4 °C and washed three times with RIPA buffer.

### 4.11. Statistical Analysis

Statistical analysis was performed by one-way analysis of variance (ANOVA) followed by Tukey’s test using Origin 9.1 (Micro Software, Northampton, MA, USA). *p*-values less than 0.05 are considered statistically significant.

### 4.12. Research Ethics

We followed biosafety guidelines for recombinant DNA research at Nagoya University. Experimental proposals were approved by the Recombinant DNA Biosafety Committee of the Graduate School of Bioagricultural Sciences, Nagoya University: Nou15-067 (approved on 24 March 2006) and Nou19-002 (approved on 12 April 2019).

## Data Availability

Not applicable.

## Abbreviations

CaM	calmodulin
CMDZ	calmidazolium
DMEM	Dulbecco’s modified Eagle’s medium
GAP	GTPase activating protein
HBSS	Hank’s balanced salt solution
HRP	horseradish peroxidase
mTORC1	mechanistic target of rapamycin complex 1
PI3P	phosphatidylinositol 3-phosphate
SOCE	store-operated Ca^2+^ entry
SUnSET	Surface Sensing of Translation
